# The P66Shc/Mitochondrial Permeability Transition Pore Pathway Determines Neurodegeneration

**DOI:** 10.1155/2013/719407

**Published:** 2013-05-15

**Authors:** Costanza Savino, PierGiuseppe Pelicci, Marco Giorgio

**Affiliations:** Department of Experimental Oncology, European Institute of Oncology, Via Adamello 16, 20139 Milan, Italy

## Abstract

Mitochondrial-mediated oxidative stress and apoptosis play a crucial role in neurodegenerative disease and aging. Both mitochondrial permeability transition (PT) and swelling of mitochondria have been involved in neurodegeneration. Indeed, knockout mice for cyclophilin-D (Cyc-D), a key regulatory component of the PT pore (PTP) that triggers mitochondrial swelling, resulted to be protected in preclinical models of multiple sclerosis (MS), Parkinson's disease (PD), and amyotrophic lateral sclerosis (ALS). However, how neuronal stress is transduced into mitochondrial oxidative stress and swelling is unclear. Recently, the aging determinant p66Shc that generates H_2_O_2_ reacting with cytochrome c and induces oxidation of PTP and mitochondrial swelling was found to be involved in MS and ALS. To investigate the role of p66Shc/PTP pathway in neurodegeneration, we performed experimental autoimmune encephalomyelitis (EAE) experiments in p66Shc knockout mice (p66Shc−/−), knock out mice for cyclophilin-D (Cyc-D−/−), and p66Shc Cyc-D double knock out (p66Shc/Cyc-D−/−) mice. Results confirm that deletion of p66Shc protects from EAE without affecting immune response, whereas it is not epistatic to the Cyc-D mutation. These findings demonstrate that p66Shc contributes to EAE induced neuronal damage most likely through the opening of PTP suggesting that p66Shc/PTP pathway transduces neurodegenerative stresses.

## 1. Introduction

The p66Shc protein is the largest isoform encoded by the ShcA locus located in the chromosome 1 in the human or chromosome 3 in the mouse genome. The ShcA locus encodes three isoforms through two different promoters, one for the p46 and p52 isoforms and the other one for the p66 isoform. Notably, the p66Shc isoform is peculiar of vertebrates being conserved in *Fucu*, *Xenophus*, *Rattus*, *Mus*, and *Homo *but not in *Saccharomyces*, *Caenorhabditis*, and *Drosophila *[1]. The p52Shc and p46Shc function as adaptor protein in signal transduction pathways linking different activated receptor tyrosine kinases to the Ras pathway. P66Shc is not involved in Ras activation, although p66Shc has the typical domain organization of all members of the Shc family of adaptor proteins. Instead, p66Shc functions in the intracellular pathways that convert oxidative signals into apoptosis [2, 3].

A fraction of p66Shc has been observed within the mitochondrial intermembrane space [4], and *in vitro *experiments revealed that the recombinant p66Shc human protein interacts and oxidizes Fe^2+^ cytochrome c to form H_2_O_2_ through a redox center that has been mapped to the N-terminus of the p66Shc protein and is missing in the other two ShcA isoforms [5]. Thus, mitochondrial p66Shc sequesters electrons from the respiratory chain to generate reactive oxygen species (ROS). 

P66Shc function is tightly regulated at multiple levels. First of all the total amount of p66Shc is regulated by transcriptional [6] and posttranslational mechanisms. In particular, the half-life of p66Shc has been demonstrated to increase upon apoptotic stimulation, notably in a p53-dependent manner [7]. The localization of p66Shc into mitochondrial intermembrane space is regulated by p66Shc posttranslational modifications including serine phosphorylation by stress kinases like Jnk-1 and Pkc-B and prolyl-isomerization by Pin-1, that induce p66Shc translocation within mitochondria [8]. 

A further level of activation of p66Shc mitochondrial function is represented by the availability of unbound p66Shc within mitochondrial vesicles. In fact, mitochondrial p66Shc has been observed to associate with a high molecular weight complex of about 670 KDa and to the mitochondrial chaperon mtHsp70. Notably, treatment of cells with proapoptotic stimuli such as Ultraviolet radiation or H_2_O_2_ induces the dissociation of this complex and thus the release of monomeric p66Shc free to react with cytochrome c [9]. Finally, the oxidation of cysteine residues and oligomerization state of p66Shc have been reported to regulate its redox function within mitochondria [10]. 

The activation of p66Shc, and the consequent H_2_O_2_ accumulation in the mitochondria, impacts on the integrity of mitochondrial inner membrane and transmembrane potential [9]. The addition of recombinant p66Shc protein to isolated mouse liver mitochondria is sufficient to induce opening of the PTP and ballooning of the vesicles [5]. The key event leading to the mitochondrial swelling is indeed the opening of the permeability transition pore (PTP), a high-conductance inner membrane channel whose molecular components have not been identified, that triggers the inner membrane permeability to solutes and as consequence to water [11].

These events are central node of cell death. During apoptosis mitochondria undergo structural and functional remodeling leading to the swelling of the organelles with the subsequent release of apoptogenic factors including cytochrome c, Smac/DIABLO, AIF, and Omi/HtrA2 into the cytosol. These factors activate a cascade of proteases responsible for nuclear DNA fragmentation and finally cell death [12]. The PTP open-closed state is regulated by multiple effectors that act on various sites [13]. In particular, it has been shown that reactive oxygen species producing by mitochondrial respiration are key regulators of PTP opening [14]. 

Consistently with the role proposed for oxidative stress on cell death and aging [15], primary mouse embryonic fibroblasts (MEFs) derived from p66Shc−/− embryos have lower intracellular concentration of ROS, as revealed by the reduced oxidation of ROS-sensitive probes and the reduced accumulation of endogenous markers of oxidative stress (8-oxo-guanosine) [7]. Likewise, p66Shc−/− mice have diminished levels of both systemic isoprostane [16] and intracellular (nitrotyrosines, 8-oxo-guanosine) oxidative stress [7]. 

Moreover, p66Shc−/− cells were shown to be resistant to apoptosis induced by a variety of different signals, including H_2_O_2_, UV, staurosporine, taxol, growth factor deprivation, calcium ionophore, osmotic shock, and CD3-CD4 crosslinking [2], and similarly different tissues of the p66Shc−/− mice were found to be resistant to apoptosis induced by paraquat [17], hypercholesterolemia [16], hyperglycemia [18], immunotoxicity [19], and ischemia [20]. 

Finally it is not surprising that p66Shc−/− mice resulted to be protected from aging-associated diseases, such as metabolic syndrome [20, 21] atherosclerosis [16], diabetes [18], and neurodegeneration [19, 22, 23] and show prolonged life span [17, 21, 22], and p66Shc/PTP pathway appears a crucial pathway of stress response involved in tissue dysfunction.

## 2. Materials and Methods

### 2.1. Cells

NG108-15, N2A, and the SH-SY5Y were grown at 37°C in 5% CO_2_ in Dulbecco's modified Eagle's medium (DMEM) the NG108-15, Modified Eagle's medium (MEM) the N2A, and the SH-SY5Y with F12 1 : 1 and 1% NeAA, supplemented with 10% (or 15% the SH-SY5Y) fetal bovine serum S.A. (Invitrogen, Carlsbad, CA, USA) and penicillin-streptomycin and glutamine (100 units/mL, 100 *μ*g/mL) (Invitrogen). 

Kelly and PC12 were cultured in RPMI 1640 medium (Gibco-BRL, Eggenstein, Germany) supplemented with 10% FBS (and 5% Horse serum the PC12) and penicillin-streptomycin (100 units/mL, 100 *μ*g/mL) (Invitrogen), under 5% CO_2_ at 37°C.

### 2.2. Western Blotting

Cells were washed twice with ice-cold PBS and lysed with 200 *μ*L of lysis buffer. Lysates were vortexed and incubated on ice for 15 min twice and then cleared by spinning at 13.000 rpm for 5 min at 4°C. Proteins were separated by 10% SDS—PAGE gels and immunoblotted according to standard western blotting procedures using primary antibody (polyclonal anti-shc, BD Biosciences).

### 2.3. Mice

P66Shc−/−, Cyc-D−/−, and p66Shc/Cyc-D−/− mice were bred in the certified IFOM-IEO campus animal facility in accordance with national and institutional guidelines. 

Mice were housed in an air-conditioned room (temperature 21 ± 1°C, relative humidity 60 ± 10%) with a white-red light cycle (lights on from 07:00 to 19:00) and with *ad libitum* food availability (2018S Teklad Global 18% Protein Rodent Diet, provided by Harlan Teklad) and drinking water (autoclaved tap water). 

All the *in vivo* experiments were performed in accordance with Italian laws and regulations. 

### 2.4. EAE

EAE was induced in p66Shc−/−, Cyc-D−/−, p66Shc/Cyc-D−/−, and C57BL/6 (Wilde type, WT) female mice (6–8 weeks of age). WT mice were obtained from Charles River (Calco, Italy) and housed in specific pathogen-free conditions, allowing access to food and water ad libitum. Procedures involving animals and their care were conducted in conformity with the institutional guidelines in compliance with national (D.L. n. 116, G.U., suppl. 40, February 18, 1992) and international laws and policies (EU Council Directive 86/609, OJ L 358, 1, December 12, 1987; Guide for the Care and Use of Laboratory Animals, U.S. National Research Council, 1996). The protocols for the proposed investigation were reviewed and approved by the Animal of the European Institute of Oncology. EAE was induced by subcutaneous immunization in the flanks with a total of 200 Ag of MOG35–55 (Multiple Peptide Systems, San Diego, CA, USA) in incomplete Freund's adjuvant (Sigma, St. Louis, MO, USA) supplemented with 8 mg/mL of *Mycobacterium tuberculosis* (strain H37RA; Difco, Detroit, MI, USA).

Mice received 500 ng of pertussin toxin (Sigma) i.v. at the time of immunization and 48 h later. Weight and clinical score were recorded daily (0 = healthy, 1 = flaccid tail, 2 = ataxia, and/or hind-limbs paresis, or slow righting reflex, 3 = paralysis of hind limb and/or paresis of forelimbs, 4 = paraparesis of fore limb, and 5 = moribund or death). The food pellets and the drinking water were placed on Petri plates on the floor of the cage to enable sick mice to eat and drink.

### 2.5. ELISA

Anti-MOG_35–55_ antibody titers were examined in the sera of the same animal groups as described previously at day 10 after immunization. Blood from these animals was obtained by heart puncture. Sera were collected and applied to MOG_35–55_-coated maxisorb 96-well ELISA plates (Nalge Nunc International, Rochester, NY) in serial dilutions. The plates were incubated for 1 h at room temperature and washed four times, and 60 *μ*L of 1 : 2500 diluted [HRP] *α*-mouse (Amersham) peroxidase conjugated mouse anti-IgG were applied to the wells. The plates were incubated for 40 min at room temperature and washed three times, and 60 *μ*L of ABTS + H_2_O_2_ substrate were applied to the wells. The reaction was performed at room temperature for 30 min in the dark. The level of reaction product was assessed as optic density (OD) at 415 nm on a standard plate reader, and the data were presented as mean_SD. OD with *n* = 3 independent measurements per group.

IL-6, IFN-*γ*, and TNF-*α* were measured in supernatants of splenocytes (see the following) by ELISA (R&D Systems, Inc.,* * Minneapolis, MN, USA) according to the manufacturers' instructions.

### 2.6. Proliferation Assay

Cells from spleens of p66Shc−/− and WT mice, stimulated or not with MOG, were cocultured for 48 h and were radiolabelled with 9,25 kBq/well of methyl-3H thymidine in the last 16 h of coculture. The cells are harvested and counted by a beta radiation liquid scintillation counter. 

## 3. Results and Discussion

### 3.1. P66Shc Determines Neuronal Cells Apoptosis

**Figure 1:**
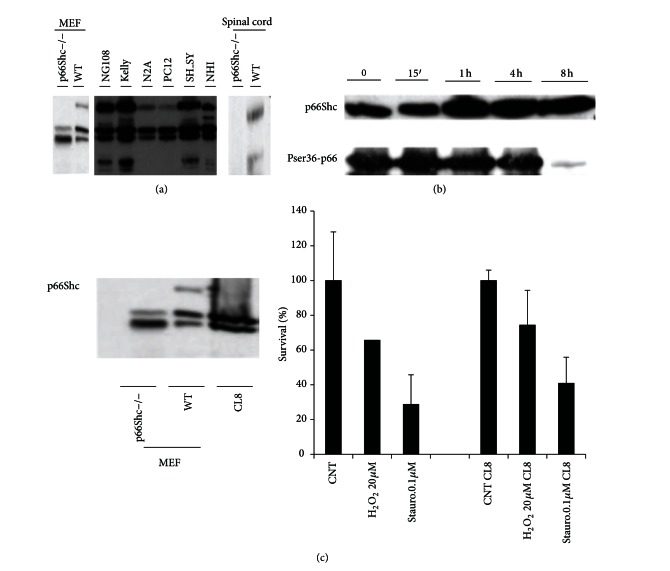
(a) P66Shc expression in different cell lines and spinal cord tissue. Western blot analysis of p66Shc was performed on whole-cell extracts from primary mouse embryonic fibroblasts (MEF), NG108, Kelly (human neuroblastoma), N2A (mouse), PC12(rat), SH-SY5Y (human neuroblastoma), NIH cells lines, and protein extract from mouse spinal cord. (b) Expression of p66Shc (upper panel) after UV treatment and (lower panel) levels of PhopsorylatedSer36-p66Shc in Kelly cells. (c) Expression of p66Shc upon specific p66Shc RNAi Interference on Kelly and response 20 *μ*M H_2_O_2_ and 0.1 mM Staurosporine.

P66Shc expression has been already demonstrated in neuronal cell lines from different mammals [23]. We confirmed by Western Blot (WB) analysis the expression of p66Shc in several neuronal cell lines, in particular Kelly and SH-NY of human origin, and in the mouse spinal cord (Figure 1(a)). The phosphorylation of p66Shc at Serine 36 is the event activating the proapoptotic function of p66Shc [8, 16]. WB analysis revealed p66Shc Ser36 phosphorylation in neuronal cell lines upon different challenges (Figure 1(b), [8]). Then, knocking down the p66Shc expression has been found to increase survival in Kelly cells (Figure 1(c)) as well as in other neuronal cells [2, 23] upon oxidative stress and other pro-apoptotic challenges. In agreement, the overexpression of p66Shc but not of the p66ShcSer36Ala mutant increased cell death in neuronal cells [23]. 

All these findings indicate that p66shc is present in neuronal cells, including human ones and confers sensitivity to apoptosis.

### 3.2. P66Shc Does Not Affect Immune Response in EAE

EAE is an acute or chronic-relapsing, demyelinating autoimmune disease model that closely resembles the human MS [24]. During EAE, as a consequence of specific myelin antigen experimental autoimmunization, activated T- and B-lymphocytes infiltrate the central nervous system (CNS) where they produce large amounts of cytokines that together with activated microglia lead to demyelination and axonal degeneration [25, 26]. Several evidences indicate that oligodendrocytes, the myelin-forming glial cells in the CNS, are sensitive to cell death stimuli such as cytotoxic cytokines, antimyelin antibodies, nitric oxide, and oxidative stress. Indeed, apoptosis has been established in glial cells of both human MS patients and EAE animal models, and it has been proposed to be the crucial mechanism of MS and EAE associated dysfunctions [27, 28].

**Figure 2:**
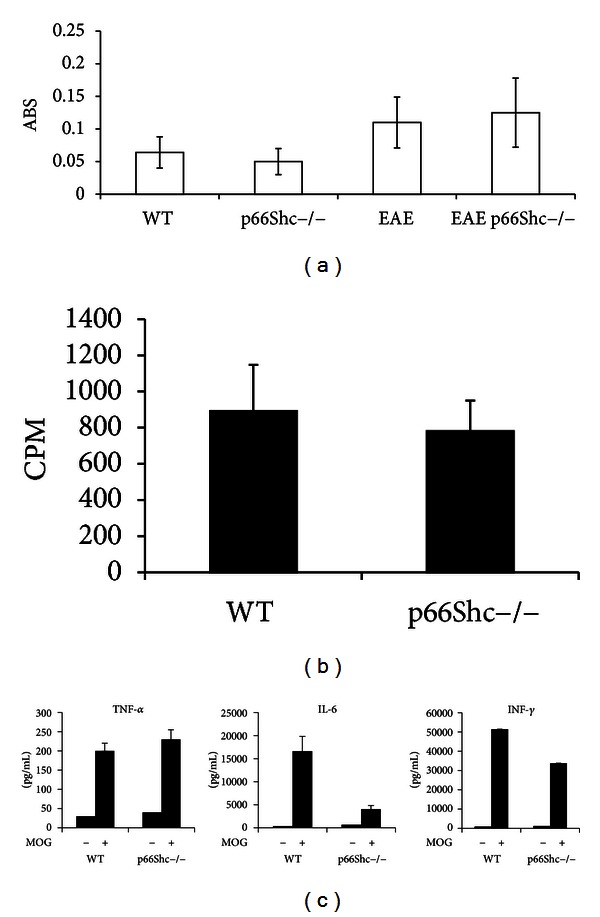
(a) ELISA assay for anti-MOG35–55 antibodies. (b) Proliferation assay. Cells from spleens, stimulated with MOG, were cocultured for 48 h and were radiolabelled with 9,25 kBq well-1 of methyl-3H thymidine in the last 16 h of coculture. The cells are harvested and counted in a liquid scintillation counter. (c) Cytokines production in medium of splenocytes from WT and p66Shc−/− mice. Results are representative of one of two experiments, each with five mice per group.

To determine the role of p66Shc in EAE we compared the results of EAE in p66Shc−/− and WT mice. First, we immunized a total number of 9 isogenic WT and 9 p66Shc−/− C57BL/6 mice with myelin oligodendrocyte glycoprotein (MOG) 35–55 peptide following standard protocols [29, 30]. At days 8, 10, and 36 upon MOG immunization, we sacrificed the mice (3 at each time) and extracted the blood and spleens to investigate anti-MOG antibodies titer and MOG-induced splenocyte proliferation, respectively. ELISA test using MOG coated wells revealed that the concentration of antibodies against MOG raised at the same time and extent in both WT and p66Shc−/− mice (Figure 2(a)). Likewise, thymidine incorporation as measure of splenocytes proliferation upon *in vitro* stimulation with MOG resulted to be not significantly altered by the deletion of p66Shc (Figure 2(b)). The secretion of TNF-alpha, IL-6, and interferon gamma by WT and p66Shc−/− splenocytes was comparable as well *in vitro* (Figure 2(c)).

Therefore, p66Shc did not appear to influence humoral and cellular immune response to MOG immunization.

### 3.3. P66Shc Deletion Delays EAE 

**Figure 3:**
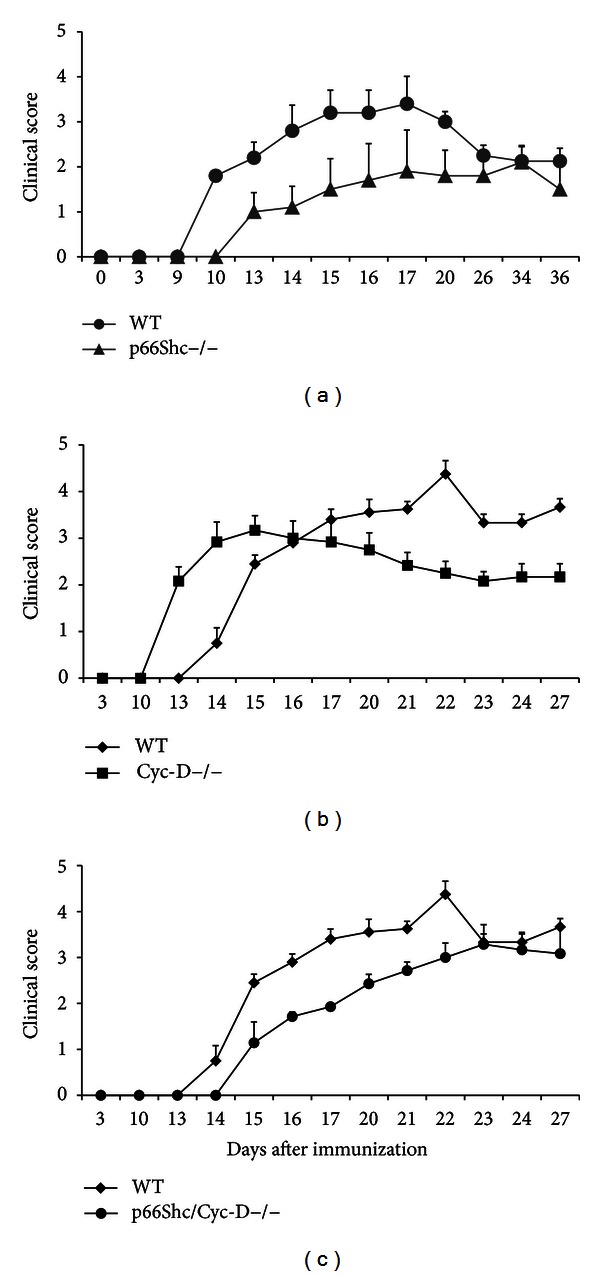
(a) EAE disease signs scores (nonparametric scale) in WT (C57BL/6) and P66Shc−/− mice have a less severe EAE disease course than WT mice. (b) EAE disease signs scores (nonparametric scale) in WT (C57BL/6) and Cyc-D−/− mice. (c) EAE disease signs scores (nonparametric scale) in WT (C57BL/6) and p66Shc/Cyc-D−/− mice.

Then we performed EAE in WT and p66Shc−/− age matched female mice. Clinical score and weight were recorded daily using a nonparametric scale. Starting from 10 days (approximately depending on the different experiments) after MOG immunization, WT mice developed clinical symptoms, manifested as limb weakness and paralysis (WT score at day 10, 1,80 ± 0,3, see Figure 3(a)) while p66Shc−/− mice did not (p66Shc−/− score at day 10, 0,0 see Figure 3(a)). The onset of the disease in p66Shc−/− mice was significantly delayed (Figure 3(a) and Table 1). All over the experiment, the p66Shc−/− showed milder paralysis than WT and consistently a lower disease severity score than WT mice (Figure 3(a)). 

Notably, the 20% EAE WT mice died, whereas no p66Shc−/− died. P66Shc deletion was found to protect significantly from body weight loss as well (day 20 body weight of p66Shc−/−  19,20 ± 1,36 versus WT 16,85 ± 1,13).

### 3.4. Cyclophilin-D Deletion Did Not Change EAE Expression in p66Shc−/− Mice

To determine whether the protective effect of p66Shc deletion was mediated by PTP, we investigated the genetic interaction between the mutations of p66Shc and of Cyc-D that increases the opening threshold of PTP and was found to ameliorate EAE as well (Figure 3(b), [31]). Indeed, the exact molecular composition of the PTP is still debated [11]. However, some putative components have been indicated, such as the voltage-dependent anion channel (VDAC) located on the outer-mitochondrial membrane, the adenine nucleotide translocase (ANT, located on the inner mitochondrial membrane), and a matrix protein, Cyc-D, but results from knockout mice of these proteins confirmed only for Cyc-D a role in mitochondrial PT, although Cyc-D−/− animals develop normally and undergo apoptosis in response to certain insults [32], suggesting that additional proteins are probably involved. 

So, we generated p66Shc/Cyc-D−/− mice by crossing p66Shc−/− and Cyc-D−/− mice both in C57BL/6 background. Then, we performed EAE on the p66Shc/Cyc-D−/− mice, and we evaluated the evolution of their disease over a month. The onset and development of EAE in p66Shc/Cyc-D−/− mice resulted to be identical to those observed for p66Shc−/− mice (Figure 3(c) and Table 2). Notably, the early onset of disease typical of Cyc-D null mice (Figure 3(b), [31]) was lost when also p66Shc was mutated suggesting that p66Shc is necessary for the expression of disease before time by the Cyc-D mutation. 

These results indicate that the deletion Cyc-D was not epistatic to the deletion of p66Shc with respect to the to EAE protection.

## 4. Conclusions

Neurodegenerative disease is complex trait disorder that involves several stress response pathways inducing mitochondrial apoptosis. Classical genetics studies, to identify susceptibility genes, are more difficult than for simple mendelian disorders because of the number of loci involved, the incomplete penetrance, and the important role of environmental factors.

Results form *in vitro* experiments showing that p66Shc increases oxidative stress to the mitochondria and the Cyc-D-dependent PTP particularly supported the hypothesis of a genetic interaction of p66Shc and Cyc-D. So, to validate the role of the p66Shc-PTP pathway in neurodegeneration we have studied in mice the epistasis of p66Shc and Cyc-D null mutations on the susceptibility to EAE.

Results from these experiments revealed that both mutations mitigated the disease, but their effects were not additive. These findings indicate that the p66Shc and Cyc-D are epistatic and validate the hypothesis that p66Shc activation by stresses converges to the PTP opening to induce mitochondrial swelling in neurons.

Therefore, early detection of p66Shc activation (expression levels, specific phosphorylation) or its inhibition may represent valid approaches to treat neurodegeneration.

## Figures and Tables

**Table 1:** Clinical EAE parameters in WT and p66Shc−/− mice.

Strain	Disease onset (day 10) score	Disease onset (day 13) score	Number of mice with score ≥3	Mean maximum score	AUC (days)
WT	1.80 ± 0.3	2.20 ± 0.34	3/5	3.20 ± 0.50	21.95 ± 3.54
P66Shc−/−	0	1.00 ± 0.43	2/5	2.5 ± 0.62	13.65 ± 4.91

Data are mean of three independent experiments ± SE. (AUC: area under the curve) values are calculated at day 20 from the immunization.

**Table 2:** Clinical EAE parameters in WT, Cyc-D−/−, and p66Shc/Cyc-D−/− mice.

Strain	Disease onset (day 11) score	Disease onset (day 13) score	Survival at sacrifice Day 27	Number of mice with score ≥3	Day of maximal score	Mean maximum score	AUC (days)
WT	0.75 ± 0.33	2.45 ± 0.18	3/10	10/10	20	4.7 ± 0.21	22.10 ± 2.91
Cyc-D−/−	0	2.08 ± 0.30	6/6	4/6	14	3.17 ± 0.31	26.83 ± 2.55
P66Shc/Cyc-D−/−	0	1.14 ± 0.37	6/7	5/7	21	3.85 ± 0.35	23.50 ± 2.24

Data are mean three independent experiments ± SE. Values are calculated at day 20 from the immunization.
